# Mr. Spry, on a Remarkable Case of Hydrocephalus Internus

**Published:** 1799-09

**Authors:** James Hume Spry

**Affiliations:** Surgeon, Aldersgate-street. London


### *To the Editors of the Medical and Phyſical Journal.*

GENTLEMEN,

SHOULD you think the following caſe of Hydrocephalus internus worthy of a place in your valuable periodical publication, by inſerting it in your next you will oblige,

Gentlemen,

Your very humble ſervaut,

JAMES HUME SPRY,

LONDON, July 16th 1799. *Surgeon, Alderſgate-ſtreet.*

Miſs

Mifs Barrow, aged four years and four months, of a delicate habit of body, and fair complexion, had always from her infancy (till her late illnefs), enjoyed a good fhare of health; having never been fubject to thofe complaints which are commonly attendant upon the infantile ftate; if we except that, during dentition, fhe had two or three convulfive fits.

About one year and a half, or two years, previous to her death, it was obferved that fhe was more than ufually drowfy; and that this drowfinefs came on, moft frequently, at the fame hour in the day: i. e. between one and two of the clock, in the afternoon, her ufual dinner-hour; it would fometimes come on a little before that time. She came to table, in general, with an appetite; but in a fhort fpace of time, often in five minutes, or lefs, would fay fhe was fleepy, and immediately, if not removed from the table, would lay her head down, and fall faft afleep. When fhe offered to take any thing, her hands were frequently obferved to tremble, or fhake; and fhe was very foon fatigued with walking: fhe was likewife obferved to eat lefs food than ufual. Being naturally of a volatile difpofition, her fpirits were generally good; and as fhe had never complained of being in the leaft indifpofed, her periodical drowfinefs was attributed by her parents to an acquired habit; confequently, the advice of a medical practitioner was never thought of.

About the 16th of May laft, fhe was feized with fymptoms of a low fever; fhe became languid, and apparently uneafy, with a fmall, quick, irregular pulfe, generally about 90 in the minute: fhe now became quite indifferent to her food, and complained of being fick with a confiderable pain in her ftomach, and likewife in her head; thefe complaints, were accompanied with coftivenefs. After taking fome aperient, and febrifuge medicines, her complaints gradually receded, till fhe was confidered as nearly recovered.

Upon the 3d of June, in the morning, the feverifh fymptoms returned; fhe became better towards noon, and was cheerful. In the evening, fhe was fuddenly feized with fymptoms of difordered vifion, as fhe complained of feeing infects flying before her eyes; almoft immediately after her making this complaint, a very violent fit of convulfions came on, which threatened every moment inftant diffolution. Her pulfe during the paroxyfm was extremely irregular; being fometimes fo quick as not to be enumerated, at other times flow, and fcarcely to be perceived; fhe remained in this ftate three hours, when fhe gradually began to recover; but the convulfive paroxyfms with delirium, recured at intervals; which were fucceeded by ftupor, and frequent fighing. She was obferved always to be worfe at the fame hour the drowfinefs ufually came on. At this period of the difeafe, her ftools became thin, of a dark clay colour, with fcybala, and were paffed involuntarily luntarily, as well as her urine; which was very high coloured, ftaining the linen of a deep yellow: when fenfible, fhe complained of the pain in her ftomach; which had gradually increafed in violence, from the firft attack. Her pulfe increafed in velocity, and became more irregular, as the difeafe advanced. During the fits of dilirium, fhe made frequent attempts to put her right hand to the pofterior part of the head; and fometimes (if not prevented) would attempt to fcratch, or tear her face. In the laft ftage of the difeafe, the fame hand and arm were affected with a conftant convulfive tremor, accompanied with vain efforts, to raife the hand to her head, which now appeared only to be affected. Near the clofe of this diftreffing diforder, her countenance underwent various, and very frequent changes; being at one time pale, at another red, and at a third of a very deep red, fimilar to a perfon fuffering ftrangulation. Her eyes at this time appeared to ftart from their orbits, with very confiderable dilatation of their pupils: coma, with convulfions at intervals, fucceeded to thefe fymptoms; and death foon clofed the fcene. She died on the 16th of June.

Various medicines were exhibited, during the courfe of the diforder, by the medical gentlemen who attended; fuch as cathartics, febrifuges, anti-fpafmodics, &c., as well as blifters; but without the fmalleft alleviation of the fymptoms.

It ought to be noticed, that two children have died in the fame family of the fame complaint; by which it would appear, that in fome families, there is a predifpofition to the difeafe: if this is really the cafe, every opportunity of infpecting the bodies of thofe children, who may be fuppofed to die of this dreadful malady, ought to be embraced by the practitioner; by which means, we may hope, at fome future time, to detect its latent caufe. But it unfortunately happens, that the prejudices of mankind are fo great againft any operation being performed upon the body of a difeafed friend or relation, as to be almoft an effectual barrier to any purfuit, or inveftigation of the kind, however great the advantages likely to be derived to pofterity by fuch an examination may be; unlefs in a few folitary inftances, where the fuperior education of the parent gets the better of the bigotry of fuperftition, the greateft enemy to the advancement of all true knowledge, and in no fcience more fo than in the fcience of phyfic.

*Diffection.*

The head was of the natural fize; and the bones were firmly united at the futures. Upon removing the upper part of the cranium, leaving the cerebrum with its membranes entire, the whole felt upon preffure uncommonly tenfe: removing the dura mater in part, leaving the falx, with the longitudinal dinal ſinuſes, the veſſels of the pia mater appeared through the tunica arachnoides, more diſtended with blood than uſual: the longitudinal ſinus was empty, probably from its poſition: the tranſverſe lateral ſinuſes were much loaded with blood: upon making an inciſion into the ventricle of the right hemiſphere of the brain; from ſix, to ſeven ounces of pellucid fluid ruſhed out with conſiderable force: the plexus choroides in each ventricle, was of a whitiſh colour: no other morbid appearance was obſerved; excepting, that the pineal gland was much more flabby than uſual.

